# Storms Are an Important Driver of Change in Tropical Forests

**DOI:** 10.1111/ele.70157

**Published:** 2025-07-09

**Authors:** Evan M. Gora, Ian R. McGregor, Helene C. Muller‐Landau, Jeffrey C. Burchfield, K. C. Cushman, Vanessa E. Rubio, Gisele Biem Mori, Martin J. P. Sullivan, Matthew W. Chmielewski, Adriane Esquivel‐Muelbert

**Affiliations:** ^1^ Cary Institute of Ecosystem Studies New York New York USA; ^2^ Smithsonian Tropical Research Institute Balboa Republic of Panama; ^3^ Department of Atmospheric Science The University of Alabama in Huntsville Huntsville Alabama USA; ^4^ Oak Ridge National Laboratory Oak Ridge Tennessee USA; ^5^ National Institute for Amazon Research (INPA) Manaus Brazil; ^6^ Programa de Pós‐graduação Em Ecologia e Conservação Universidade Do Estado de Mato Grosso Nova Xavantina Brazil; ^7^ Department of Natural Sciences Manchester Metropolitan University Manchester UK; ^8^ Department of Biology University of Louisville Louisville Kentucky USA; ^9^ School of Geography, Earth and Environmental Sciences University of Birmingham Birmingham UK; ^10^ Birmingham Institute of Forest Research University of Birmingham Birmingham UK

**Keywords:** biomass carbon, carbon cycling, carbon sink, climate change, convective storms, drought, forest dynamics, tree mortality, vapour pressure deficit (VPD)

## Abstract

Tropical forest dynamics and composition have changed over recent decades, but the proximate drivers of these changes remain unclear. Investigations into these trends have focused on increasing drought stress, CO_2_, temperature, and fires, whereas convective storms are generally overlooked. We argue that existing literature provides clear support for the importance of storms as drivers of forest change. We reanalyze the largest plot‐based study of tropical forest carbon dynamics to show that lightning frequency—an indicator of storm activity—strongly predicts forest carbon storage and residence time, and its inclusion improves model fit and weakens evidence for the effects of high temperatures. Convective storm activity has increased 5%–25% per decade over the past half century. Extrapolating from historic trends, we estimate that storms likely contribute ca. 50% of the reported increases in biomass mortality across Amazonia, with all realistic combinations of assumptions indicating a possible range of 12%–118%. Spatial variation in storm activity shows weak relationships with drought, demonstrating that forests can experience high drought stress, high storm activity, or both. Accordingly, we hypothesise that convective storms are among the most important drivers of tropical forest change, and as such, they require significant research investment to avoid misguiding science, policy, and management.

## Graphical abstract

This paper presents literature syntheses and re‐analyses of published data showing that convective storms (i.e., not hurricanes) are a dominant driver of biomass carbon losses in tropical forests. Increases in storm activity during the past half century are likely a major contributor to ongoing patterns of change among tropical forests, including the weakening of the tropical forest carbon sink. We conclude that there is very similar evidence for both storms and drought stress as drivers of forest change, and we recommend that storm‐caused mortality receive equal consideration alongside other potential drivers of forest change.

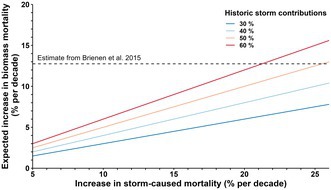

Tropical forest dynamics are changing with major implications for biodiversity, carbon storage, and global climate (Bauman et al. 2022; Brienen et al. 2015; Esquivel‐Muelbert et al. 2019; Fadrique et al. 2018; Feeley et al. 2020). Although human‐driven deforestation and degradation are the primary threats to tropical forests (Gatti et al. 2021; Lapola et al. 2023; Pan et al. 2024; Qin et al. 2021), even intact forests unaffected by deforestation or degradation are rapidly changing. Tropical tree mortality rates within intact forests have increased in many regions over recent decades (Bauman et al. 2022; Brienen et al. 2015; Qie et al. 2017), although not everywhere (Hubau et al. 2020; Rutishauser et al. 2020), and tree species composition is shifting (Cuni‐Sanchez et al. 2024; Esquivel‐Muelbert et al. 2019; Fadrique et al. 2018; Feeley et al. 2020). These changes among intact tropical forests are generally attributed to changes in climate and/or atmospheric CO_2_ concentrations (McDowell et al. 2018), which implies these changes will persist for centuries even if we stop deforestation and greenhouse gas emissions (Mason‐Delmotte et al. 2022). Because different drivers of forest change have different implications for future forest trajectories (Gora and Esquivel‐Muelbert 2021; McDowell et al. 2020), it is imperative that we identify and quantify the key climate‐associated drivers of forest change to inform forest management, guide successful reforestation efforts, and develop realistic Earth system models to guide policy (Friend et al. 2014; Koch et al. 2021; McDowell et al. 2020).

The causes of changing tree mortality trends remain unclear despite substantial research effort. Research into climate‐driven tree mortality has primarily focused on the roles of periodic drought, temperature, fire, CO_2_, and vapour pressure deficit (VPD) (Balslev et al. 2022; Brando et al. 2019; McDowell et al. 2020). This work has identified promising associations (Bauman et al. 2022; Tavares et al. 2023) and explained some spatial variation in mortality (e.g., explaining 31% of pantropical variation in plot‐measured biomass carbon residence time; biomass carbon residence time equals one divided by the biomass mortality rate) (Sullivan et al. 2020). However, trends of increasing tree mortality, weakening of the carbon sink in intact tropical forests, and shifting forest composition remain largely unexplained (Esquivel‐Muelbert et al. 2019; Hubau et al. 2020). Our limited ability to explain these trends could be in part because we are omitting key drivers. This raises an important question: Are there equally plausible alternative hypotheses that we are not testing?

In this perspective, we demonstrate that convective storms are an important agent of change in tropical forests. Our argument has four parts. First, we summarise the evidence for the importance of convective storms as a major, climate‐sensitive agent of tree death and a likely driver of forest change (Negrón‐Juárez et al. 2018; Yanoviak et al. 2020). Second, we synthesise the literature investigating climate‐driven change among tropical forests, placing convective storms in this context. Third, we show that strong effects of storms and drought stress are not mutually exclusive. Finally, we discuss why storm‐caused mortality is challenging to quantify and highlight avenues for needed advances in this field. Overall, we conclude that there is very similar evidence for both storms and drought stress as drivers of forest change, and we recommend that storm‐caused mortality receive equal consideration alongside other potential drivers of forest change.

## 1 The Case for Convective Storms as a Key Agent of Climate‐Driven Forest Change

**FIGURE 1:**
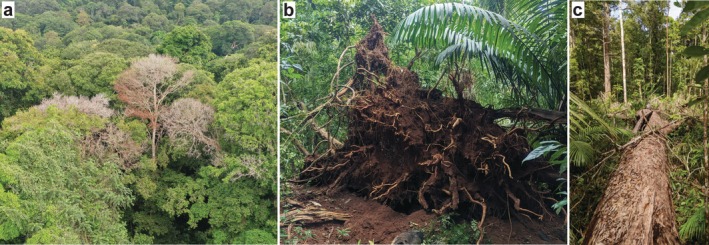
Storms kill groups of trees via lightning and wind. Example images of tree mortality caused by lightning (a), wind‐caused uprooting (b), and the collateral damage caused by a fallen large tree (c). Images taken on Barro Colorado Island in Panama (a and b) and in Reserva Florestal Adolpho Ducke in Brazil (c) by E.M. Gora.

Convective storms are the dominant drivers of tree biomass mortality in many tropical forests, killing trees primarily via windthrow and lightning (Figure 1). Convective storms are defined here as storms with vertical instability associated with strong winds and lightning that are typically small in scale (10 s or 100 s of km^2^) and, for this perspective, we consider them separately from large cyclonic systems like hurricanes or typhoons. Nearly all storm‐associated mortality events are small (> 98% of events are < 0.1 ha) (Amir 2012; Anderson 1964; Araujo et al. 2021; Brünig 1964; Chambers et al. 2013; Furtado 1935; Gora et al. 2021; Sherman et al. 2000), yet small events like these contribute the vast majority of biomass mortality in tropical forests, including > 98% of biomass mortality across the Amazon (Espírito‐Santo et al. 2014; Jackson, Fischer, et al. 2024). However, the contributions of individual mortality agents, including storms, to tropical forest biomass mortality remain unquantified (McDowell et al. 2018).

Existing data suggest that wind and lightning combine to cause at least half of pantropical biomass mortality in forests. Storm‐associated winds break and uproot canopy trees, which themselves damage and kill additional trees when they fall (Fontes et al. 2018). Forest inventories in the Amazon estimated that 51% of all trees die broken or uprooted, a large majority of which is apparently caused by wind (Esquivel‐Muelbert et al. 2020; Fontes et al. 2018). Given its disproportionate effects on large trees (Gora and Esquivel‐Muelbert 2021), this data suggest that wind causes approximately half of Amazonian biomass mortality, with a highly conservative range of 25%–50%. Lightning strikes damage and kill groups of standing trees in tropical forests, on average damaging 24 and killing 5 trees per strike without causing fire or explosive damage (we note that lightning is not a common ignition source for tropical forest fires) (Gora et al. 2021; Gora and Yanoviak 2020; Yanoviak et al. 2020). Lightning‐associated mortality contributes at least 16% of biomass turnover in the only site where its contribution has been quantified, albeit in a forest with high lightning frequency (Gora et al. 2021). Based on similar observations of per‐strike lightning damage across the tropics (Gora and Yanoviak 2020), we expect that the 35–67 million lightning strikes hitting tropical forests every year cause ca. 5%–10% of pantropical biomass mortality (Gora et al. 2020). Overall, wind and lightning kill billions of trees each year (Chambers et al. 2013; Gora et al. 2020; Negrón‐Juárez et al. 2018) with disproportionate effects on large trees, and based on existing data, we conservatively estimate that storms cause 30%–60% of pantropical biomass mortality.

**FIGURE 2:**
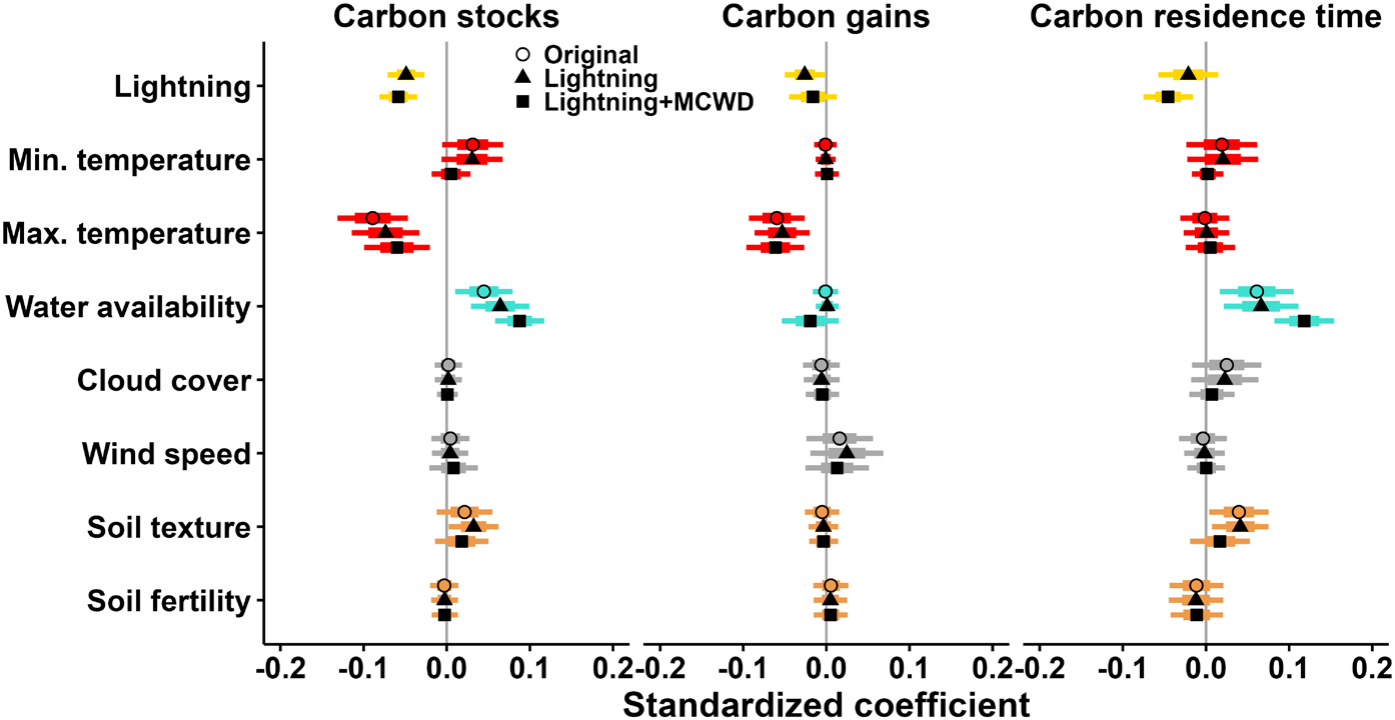
Storm activity, estimated as lightning frequency, predicts tropical forest carbon. Points represent model‐averaged coefficients of major drivers of biomass carbon stocks, productivity (‘gains’), and carbon residence time in intact tropical forest. The points show values for the original model (open circles; (Sullivan et al. 2020)), this model with lightning frequency added (triangles), and the overall best‐fit model that included lightning and also replaced precipitation in the driest quarter with maximum climatological water deficit, or MCWD, as the measure of water availability (squares). Thick bars represent standard error of each coefficient, whereas thinner bars represent their 95% confidence intervals. See Table S1 for detailed model results.

Storm activity also predicts spatial and temporal patterns of forest disturbance rates, forest structure, and aboveground biomass carbon, as would be expected if storms are a major driver of variation in tropical biomass mortality rates. Lightning strike frequency, convective available potential energy (CAPE), wind speed, and rainfall rate are all used as proxies for convective storm activity in such analyses (Araujo et al. 2021; Feng et al. 2023; Gora et al. 2020; Gorgens et al. 2021; de Lima et al. 2023). A pantropical analysis showed that forests with higher lightning frequency have fewer large trees, higher rates of annual biomass mortality, and less total aboveground carbon than forests with lower lightning frequency (Gora et al. 2020). Similarly, among Amazonian forests, those that experience more lightning strikes and stronger convective winds have shorter maximum heights (Gorgens et al. 2021) and lower taxonomic diversity among large trees > 70 cm in diameter (de Lima et al. 2023). Data connecting temporal patterns of storm activity to forest dynamics are almost non‐existent, but a unique 5‐year study in Panamanian lowland forest found that the frequency of 15‐min periods of extreme rainfall, which are a proxy for strong convection, was the best predictor of monthly variation in canopy disturbance rates (Araujo et al. 2021). In Box 1 and Figure 2, we present a reanalysis of the largest plot‐based study of forest biomass carbon, showing that lightning frequency, temperature, and water availability are similarly important predictors of pantropical patterns of forest biomass carbon storage, productivity, and residence time. Including lightning improved model fit and reduced estimates of high temperature effects, particularly in the hottest forests. Collectively, these patterns provide strong evidence that convective storm activity is a key factor shaping spatial and temporal trends in tropical forest composition, structure, carbon storage, and disturbance rates.

BOX 1 **Linking convective storms to pantropical biomass carbon dynamics.**

Here, we show that storm activity is a strong predictor of tropical forest carbon cycling. Specifically, we included lightning frequency (estimated by a global network of sensors, see Gora et al. (2020) for details about the lightning data) in a reanalysis of the ForestPlots.net dataset published by Sullivan et al. (2020), which is, to our knowledge, the largest plot‐based pantropical dataset of biomass carbon stocks and fluxes (637 ha across 590 plots). We use lightning as a proxy for storm activity because it is associated with damaging winds (Williams et al. 1999) and it almost exclusively occurs during convective storms (Williams 2005).

The original study found that maximum temperature and precipitation in the driest quarter of the year were the most important predictors of carbon stocks: higher maximum temperature was associated with lower productivity and lower carbon stocks, and less precipitation with shorter carbon residence time and lower carbon stocks (Figure 2). These effects on carbon stocks persist, but the effect of temperature weakens when lightning is included; lightning substantially improves model fit (including lightning decreases AIC by 17.2) and the effect of lightning is similar in magnitude to that of maximum temperature and precipitation in the driest quarter (Figure 2 and Table S1). Higher lightning frequency reduces carbon residence time and carbon stocks.

The best‐fit model included lightning together with water availability (maximum cuwater availability, or MCWD) rather than precipitation in the driest quarter, and exhibited an altered shape of the relationship between carbon stocks and temperature. MCWD is a more direct proxy of water limitation than precipitation in the driest quarter. The original analysis found that the best‐fit model for forest carbon stocks included a temperature by precipitation interaction and a breakpoint temperature effect of 32°C, above which temperature had a stronger negative effect on carbon stocks (AIC equaled 60.9 and 75.6 for the breakpoint and linear models, respectively) (Sullivan et al. 2020). However, the lower carbon stocks in the hottest forests, which are located in the southern Amazon and also experience high storm activity (Figure 4), could alternatively be explained by a model with lightning and its interactions with temperature and MCWD (instead of precipitation in the driest quarter). This model provides a better fit to the carbon stock data (AIC = 37.7) than the original model, and it does not support a breakpoint effect for temperature. Thus, the low carbon stocks in the hottest forests could be attributed to the combined effect of storms, temperature, and moisture availability, rather than being attributed mainly to extreme temperature.

Convective storms are increasing in frequency across tropical forests. From 1975 to 2017, the number of days during which thunder was recorded by meteorological stations, which is a strong proxy for lightning frequency and storm activity, more than doubled across the Amazon and increased by 20%–50% across central America, the Congo Basin, and India (Lavigne et al. 2019). Additionally, satellite‐based measurements of cloud height and extent captured strong increases in the intensity and extent of thunderstorms across the Congo Basin from 1982 to 2016 (Raghavendra et al. 2018). This finding was corroborated by a hindcasting study based on strong empirical relationships of thunderstorm activity with specific humidity and vertical air flow; this study estimated that thunderstorm activity increased ca. 30% over Africa during 1950–2015, with the largest increases since 1990 and over the Congo Basin (Harel and Price 2020). Moreover, afternoon CAPE predicts spatial variation in large‐scale windthrow events (Feng et al. 2023), and both afternoon CAPE and large‐scale windthrow events have increased across the Amazon from 1985 to 2020 (Urquiza‐Muñoz et al. 2024). CAPE and thus thunderstorm activity are projected to increase substantially in coming decades, although temporal trends in CAPE are highly uncertain (Feng et al. 2023; Taszarek et al. 2021). These trends from diverse sources of data all suggest that convective storm activity has increased by 5%–25% per decade over the past half‐century and that these increases are likely to continue (Harel and Price 2020; Lavigne et al. 2019; Raghavendra et al. 2018; Urquiza‐Muñoz et al. 2024).

**FIGURE 3:**
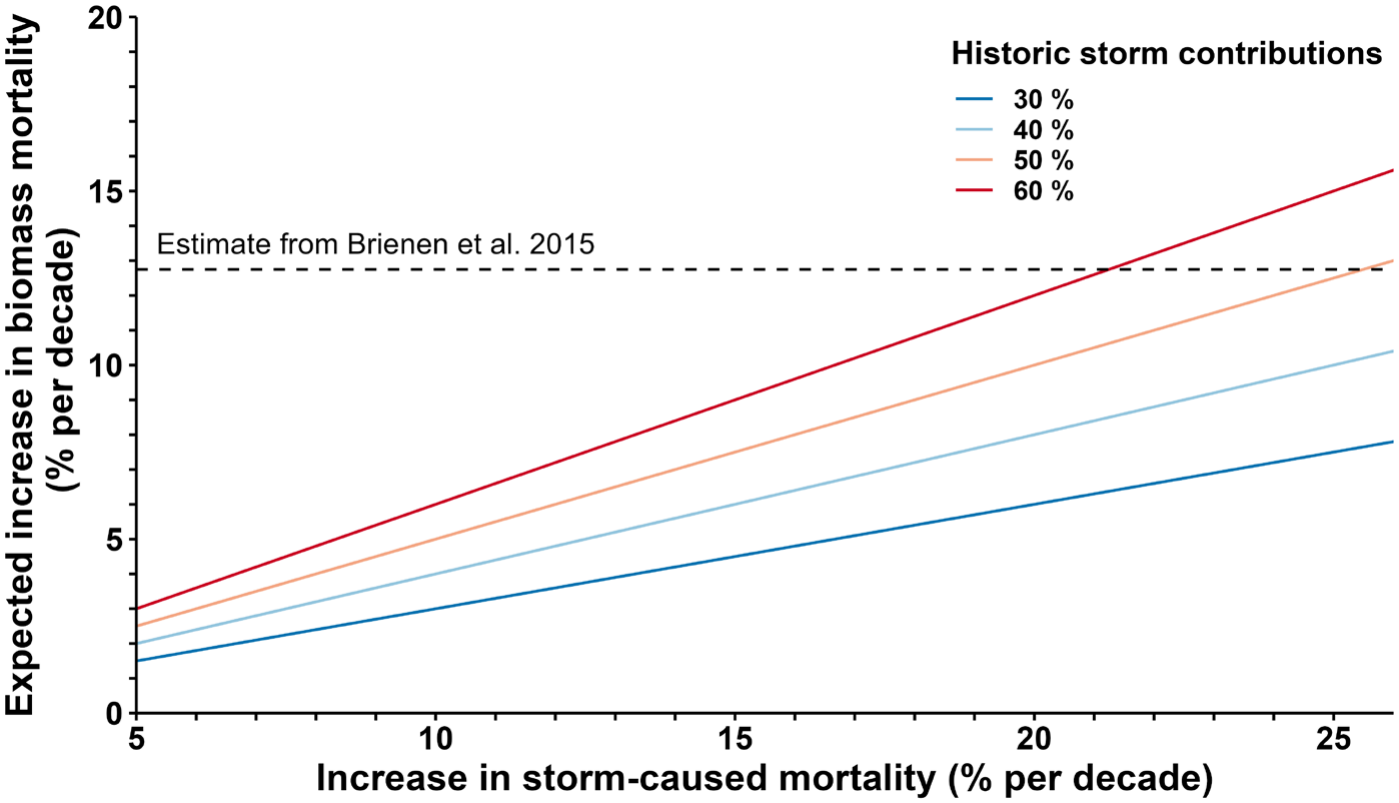
The expected increase in biomass mortality due to increasing storm activity. We estimate the expected increase in biomass mortality (*y*‐axis) for a range of potential increases in storm‐caused mortality (*x*‐axis) and differing assumptions about the contributions of storms to historic, baseline biomass mortality (different coloured lines). The range of increases in storm‐caused mortality shown here reflects the range of estimated increases in storm activity over the past half century for different studies and different tropical regions (see main text). For reference, the horizontal dashed line shows the increase in biomass mortality reported for the Amazon for 1990–2011 by Brienen et al. (2015), which is the strongest evidence for increasing biomass mortality in intact tropical forests. As described in the main text, the limited existing data suggest that storms are the proximate causes of at least 50% of historic biomass mortality, implying that the orange and red lines provide the most realistic estimates.

Observed increases in storm activity in tropical forests could explain much of the observed increase in biomass mortality in tropical forests, and associated contributions to weakening of the tropical carbon sink. To illustrate this point, we calculate the expected increases in forest biomass mortality for a range of increases in convective storm effects (5%–25% per decade) and for alternative assumptions regarding the historic contributions of convective storms to biomass mortality described above (30%–60%; Figure 3). We compare these expected increases to the observed increase in biomass mortality reported for the Amazon (Brienen et al. 2015). Depending on the exact assumptions, increasing storm activity can account for 12%–118% of the reported increase in biomass mortality (Figure 3). Based on the literature described above, a moderate estimate is that storms contribute 50% of historic biomass mortality and that storm‐caused mortality is increasing 15% per decade (see references above), which would cause biomass mortality to increase ~7% per decade (see orange line in Figure 3), thus accounting for a little more than half the increase reported by Brienen et al. for the period of 1990–2011 (Brienen et al. 2015). This estimate is highly imprecise and does not account for possible nonlinearities related to potential mitigating effects of forest acclimation to increasing storm frequency or potential amplifying effects of interactions with other drivers (Gora and Esquivel‐Muelbert 2021). However, regardless of the precise estimate, all realistic combinations of assumptions result in the conclusion that convective storms are a major contributor to climate‐driven change in tropical forest dynamics.

## 2 How Do Storms Fit Into Our Current Understanding of Climate‐Driven Tropical Forest Change?

Storms are only one of multiple potential drivers of climate‐associated change in tropical forests. Contemporary forest woody productivity generally exceeds biomass mortality in intact tropical forests, producing a sink that is likely due to CO_2_ fertilisation (Fernández‐Martínez et al. 2019; Haverd et al. 2020; Hubau et al. 2020). At the same time, increases in temperature and deficits of precipitation can cause heat and drought stress that decrease forest productivity and contribute to temporary reversals of the tropical forest carbon sink (Brando et al. 2019; Liu et al. 2017; Slot and Winter 2016). Drought stress also causes tree mortality via high atmospheric demand (high vapour pressure deficit or VPD), low ground water supply (high MCWD), or a combination of both (i.e., ‘hot drought’) (Hammond et al. 2022). Spatial variation in drought stress is strongly related to species distributions (Condit et al. 2013; Esquivel‐Muelbert et al. 2017), and drought events in tropical forests typically increase tree mortality rates by 1%–5% (Phillips et al. 2010). Consequently, increases in drought stress could be causing increased tree mortality, in addition to reduced productivity (Barkhordarian et al. 2019; Bauman et al. 2022; Boisier et al. 2015; Brando et al. 2019; Duffy et al. 2015; Tavares et al. 2023; Trenberth et al. 2014) and altered species composition (Esquivel‐Muelbert et al. 2019), but the contributions of drought stress to climate‐driven increases in tropical tree death remain highly uncertain.

Increasing VPD is a strong candidate for explaining observed patterns of forest change (Barkhordarian et al. 2019). Temperatures are increasing across the tropics; higher temperatures inherently increase VPD, all else equal, and higher VPD increases drought stress (Grossiord et al. 2020; Smith et al. 2020). Indeed, the strong effects of high temperatures on forest carbon cycling described in Box 1 (Sullivan et al. 2020) are likely due, at least in part, to the effects of high VPD on tree growth. Moreover, VPD was associated with a doubling of tree mortality rates over 49 years across 13 ha of plots in northern Australia, with the most affected species located at the upper end of their VPD range (Bauman et al. 2022). These patterns are compelling, but they are fundamentally correlative, and VPD‐caused mortality is challenging to confirm and quantify. Given that increased temperature and VPD are general proxies for the magnitude of climate change, it is likely that their effects occur in parallel with other climate‐driven agents of mortality. Indeed, storm activity also tends to increase with higher temperatures (Romps 2019; Romps et al. 2014) and has increased alongside VPD during recent decades (Harel and Price 2020; Lavigne et al. 2019; Raghavendra et al. 2018; Urquiza‐Muñoz et al. 2024).

Among all hypothesized agents of tropical forest change, periodic droughts have received perhaps the most research attention. Forest plots have captured increased tree mortality and decreased growth during census intervals including periodic droughts (Phillips et al. 2009; Qie et al. 2017) (but see (Bennett et al. 2021)), and satellite data have demonstrated increased drying and decreased greenness during droughts (Chen et al. 2024; Saatchi et al. 2013). Moreover, across 10 Amazonian sites, communities with lower mean hydraulic safety margins (the difference between species drought tolerance and observed dry season hydraulic stress) had higher stem mortality rates and lower net biomass change (Tavares et al. 2023). However, nearly all relevant studies contrast drought versus non‐drought years without demonstrating a temporal trend in drought effects, and attempts to explain increasing rates of biomass mortality among forest plots do not find a statistical link with increasing drought over time (Brienen et al. 2015; Hubau et al. 2020; Tavares et al. 2023). These data and much additional research not cited here show that droughts can cause forest change and are likely contributing to shifts in forest dynamics (reviewed by (Brando et al. 2019; McDowell et al. 2018)), but their limited explanatory power suggests that other drivers likely play key roles.

Quantifying the importance of drought is further complicated by variation in forest resilience and confounding drivers of forest change. Droughts associated with the extreme 2015–2016 El Niño event increased mortality in the Amazon but not in Africa (Bennett et al. 2021), and experimental droughts increased tree mortality in Amazonia but not in Australia (da Costa et al. 2010; Nepstad et al. 2007; Pivovaroff et al. 2021). In the Americas, the largest increases in carbon emissions are found in the southeastern Amazon, which is notably hot and dry, but also the area suffering the most deforestation, degradation, and associated fires (Gatti et al. 2021). Moreover, observational studies quantifying the effects of periodic drought events struggle to confidently separate their contributions from other drivers. For example, the 2005 drought in Amazonia coincided with elevated storm activity in the same year (Negrón‐Juárez et al. 2010; Phillips et al. 2009), and the only study to investigate monthly mortality during this timeframe found an increase in mortality during the preceding wet season rather than the drought (Aleixo et al. 2019). We do not suggest that drought did not cause meaningful mortality in 2005 or in any other drought year. However, the data indicate that coincident storm and drought effects can be confounded in plot‐based studies, possibly leading to an overestimation of drought‐caused mortality and making the use of these data for validation of satellite trends problematic (Saatchi et al. 2013).

The argument for storms as an agent of climate‐driven change parallels the argument for drought and VPD as drivers of increased tree death. Spatial variation in storm activity, like spatial variation in climatic water deficits, is a strong correlate of spatial variation in forest biomass, biomass mortality rates, and species composition (Gora et al. 2020; Gorgens et al. 2021; de Lima et al. 2023) (Box 1). Temporal variation in storm activity, like temporal variation in drought stress, predicts temporal variation in tree mortality rates (Araujo et al. 2021). Storm frequency in the tropics, like drought magnitude and frequency, has increased in parallel with increasing tropical tree mortality rates over the past several decades (Harel and Price 2020; Lavigne et al. 2019; Raghavendra et al. 2018; Urquiza‐Muñoz et al. 2024). There are two major differences between storms and drought stress. First, storms do not meaningfully influence productivity, so unlike drought stress, their importance is exclusively due to their effects on tree damage and death. Second, storms cause a large proportion of historic biomass mortality (Gora et al. 2020, 2021; Gora and Esquivel‐Muelbert 2021; Negrón‐Juárez et al. 2018) (Box 1), so even a small relative increase in storm effects could cause a large change in forest dynamics. Given the relatively strong evidence for storm‐caused forest change, the limited research effort into convective storms is notable. We do not argue that non‐storm factors are unimportant; however, the quantitative contributions of non‐storm drivers to changing forest dynamics are unclear, and storms could be similarly or even more important.

**FIGURE 4:**
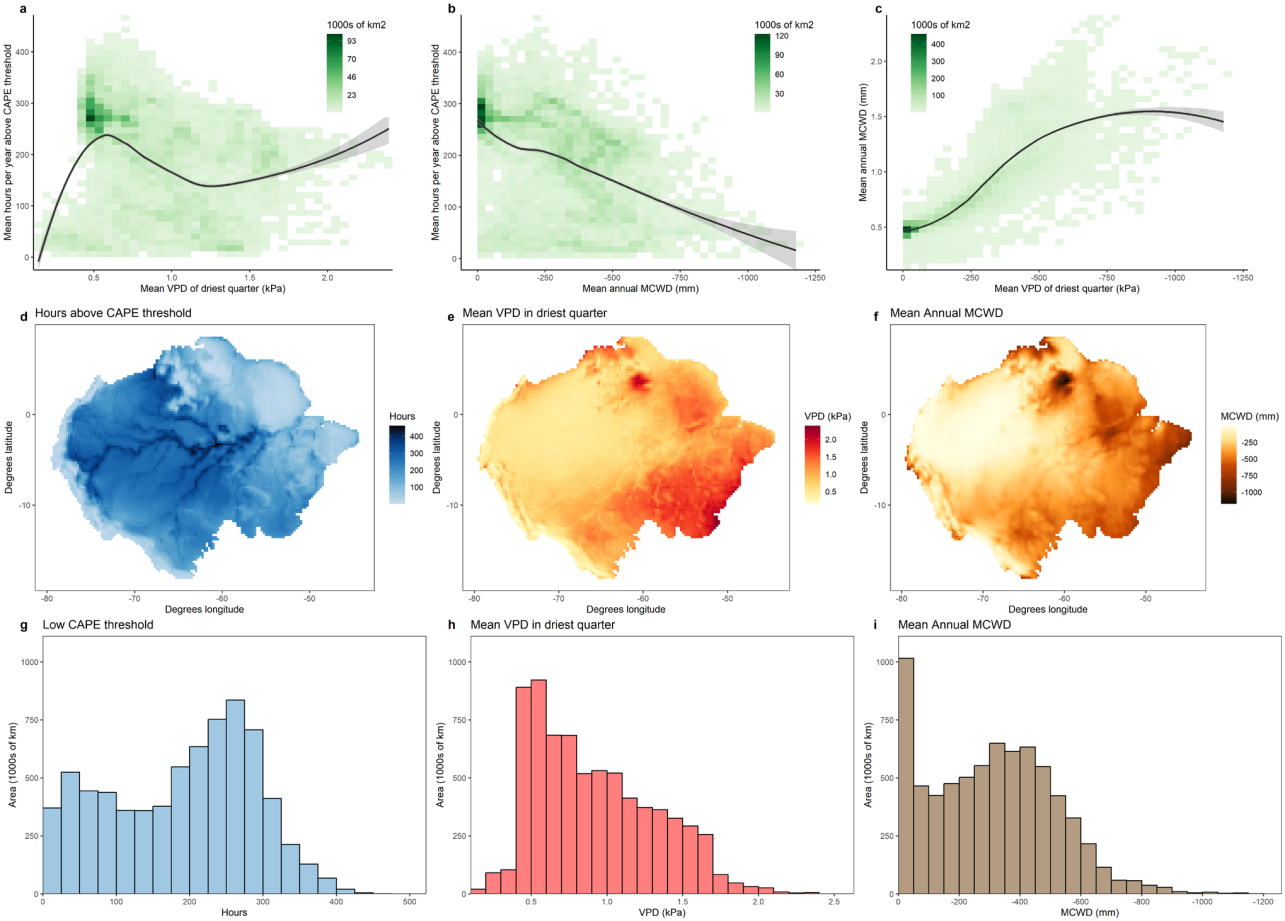
Storm activity and drought stress are weakly associated across the Amazon region. The amount of time that forests experienced levels of CAPE above a general threshold for convection (greater than the 75th percentile of CAPE or 1023 J kg^−1^) was largely unrelated to VPD in the driest quarter of the year (VPD averaged across the 3 consecutive months of highest VPD values) (panel a) and exhibited a weak negative relationship with mean annual MCWD (panel b), with a trade‐off between storm activity and MCWD only at their highest values. Mean annual MCWD and VPD in the driest quarter were strongly positively associated (panel c). These values were averaged over years 1990–2019. The same data are shown as histograms of forest area (panels g‐i) and maps of the Amazon (panels d‐f), including Andean and Guiana Shield forests, with the annual average number of hours with CAPE above this threshold for convection (panels d and g), VPD in the driest quarter (panels e and h), and mean annual MCWD (panels f and i). Shading in panels a–c represents total forest area that experiences those climatic conditions, whereas colours in panels d‐f represent variation in each metric over space. We focus this analysis on present‐day spatial patterns within the Amazon region because it has exhibited the strongest evidence of forest change, likely because it is comparatively well studied, and temporal trends in existing CAPE products are poorly constrained (Taszarek et al. 2021).

It is commonly believed that storm activity is low in drier forests because they receive less rain, but the relationships between drought stress and storm activity are weak and inconsistent. Climate reanalysis products (European Centre For Medium‐Range Weather Forecasts 2019) show essentially no relationship between convective activity (here measured as time above the CAPE threshold needed to produce convection) and maximum annual aridity of the atmosphere across Amazonia (peak aridity was estimated as VPD in the driest quarter based on the relationship between this metric and increased tree mortality in northern Australia; Figure 4a; (Bauman et al. 2022)). The only exception to this trend is coincident low VPD and low convective activity in higher elevation Andean forests. Additionally, there is a negative association between CAPE and mean annual MCWD due to a trade‐off between extreme CAPE (12% of forest area with > 300 h year^−1^ above the CAPE threshold) and extreme MCWD (18% of forest area with mean annual MCWD above −500 mm), but below these thresholds, the vast majority of forest area experienced moderate‐to‐high storm activity and a wide range of mean annual MCWD (0–600 mm) (Figure 4b). This contrasts with the strong correspondence between MCWD and VPD (Figure 4c), showing that forests with low water supply consistently experience high aridity. Moreover, convective activity is very high across the southern Amazon where drought stress is also high and patterns of change are most extreme (Gatti et al. 2021) (Figure 4d–f). Overall, these patterns show that the effects of aridity and convective activity are not mutually exclusive, and it is plausible that increases in drought stress are co‐occurring with increases in storm activity.

## 3 Why Are Convective Storms Rarely Considered as an Agent of Climate‐Driven Change?

Investigations of convective storms and drought stress present different challenges. Because of their distinct mechanisms of action, there are fundamental differences in their frequency, severity, spatial extent, and degree of aggregation. Specifically, severe droughts cause a periodic 1%–5% increase in tropical tree mortality rates across a large area once or twice a decade (Chen et al. 2024; Phillips et al. 2010), whereas storm‐caused disturbances increase tree mortality rates by 2%–80% and typically cause > 50% biomass mortality in clusters of small‐scale disturbances (< 0.1 ha) every year (Cushman et al. 2021; Gora et al. 2021; Negrón‐Juárez et al. 2018; Rifai et al. 2016; Silvério et al. 2019; Terborgh et al. 2020). Consequently, commonly available data are better suited for detecting drought stress than convective storms.

Drought stress is easily evaluated over large spatial scales. The timing and severity of drought stress events are readily quantified with common meteorological data (particularly rainfall and temperature), and they are directly perceived by humans. In most tropical forests, high drought stress occurs simultaneously across large expanses once or twice a decade in association with the ENSO cycle. Thus, the vast majority of trees in a region experience drought stress at the same time, and the strong periodicity and synchrony of drought stress allow easy comparison to non‐drought stress years at the same site. These characteristics make drought stress effects relatively easy to detect with forest plots despite their limited spatial extent, and with satellite data despite the challenges of detecting small‐scale events, although quantification of drought‐caused mortality remains challenging for the reasons described above.

By contrast, storm‐caused disturbances are comparatively challenging to detect. Unlike drought stress, only a small proportion of forest area experiences storm‐caused disturbance in even the stormiest years, with the importance of storms derived from their high severity rather than their spatial coverage. Individual lightning strikes are cryptic disturbances that are generally unidentified as such because this requires both high‐frequency monitoring and field teams trained in the identification of “flashover” damage, which are a rare combination (but see (Gora et al. 2021; Gora and Yanoviak 2020; Yanoviak et al. 2020)). Wind‐caused disturbance is easier to identify than lightning if it is observed shortly after the event (i.e., before decomposition obscures whether a tree began decomposing before or after it fell), but it is often overlooked as a stochastic “background” event. Satellite data have revealed much about the role of large‐scale windthrow in forest dynamics (Feng et al. 2023; Negrón‐Juárez et al. 2018, 2010), but the small events (< 0.1 ha) that constitute nearly all storm‐associated biomass mortality are not yet reliably detected using satellite imagery (Araujo et al. 2021; Cushman et al. 2021; Gora et al. 2021; Jackson, Fischer, et al. 2024). Improvements in field and satellite methods could overcome these issues in the future, but storm‐caused disturbance is often unattributed using existing methods.

In addition to being small in area, individual windthrows and lightning strikes are challenging to quantify because they are highly aggregated in space and time. The only site where lightning strikes and wind‐associated treefall events were monitored with sufficient frequency and scale to estimate their degree of aggregation (monthly or continuous monitoring of 50‐ha) showed that 35% of all lightning strikes and 31% of wind‐disturbed area (which was 22.6% of the total canopy disturbance area) occurred during a single month of a 5‐year period, with most of these occurring during a single storm event on a single day (Araujo et al. 2021; Gora et al. 2021). The high aggregation of these events means that quantifying an increase in storm‐associated mortality would require measuring storm‐caused mortality across hundreds or even thousands of hectares, depending on the timeframe and methods of monitoring (McMahon et al. 2019).

## 4 Future Directions

There remains substantial uncertainty about the contributions of storms to current and future forest dynamics. Storm effects are challenging to study in the field, as described above, and storm‐caused mortality primarily occurs during localised extreme events whose frequency and intensity have little‐to‐no relationship with factors that are often invoked as predictors of storm damage, such as mean wind speed or total precipitation (e.g., (McDowell et al. 2018; Sullivan et al. 2020)). In addition, we have little knowledge or data about the physiological processes or traits that control tolerance to lightning (Gora et al. 2017; Richards et al. 2022) and wind among tropical trees (Jackson et al. 2021; Jackson, Bittencourt, et al. 2024), so we have little basis for predicting how increasing storms will shift plant community composition or function. There is also a disconnect between the processes regulating the generation of storms, which operate at continental scales and decadal timelines (Dowdy 2016; Mann et al. 1995), and the small scale (< 0.1 ha) of the storm‐caused mortality events that dominate trends in tree mortality (Chambers et al. 2013; Espírito‐Santo et al. 2014; Gora et al. 2021; Negrón‐Juárez et al. 2018). Ultimately, we need better data quantifying storm‐caused disturbance in the field, assessing the physiological and anatomical traits that control tolerance to wind (e.g., elasticity, wood density, modulus of rupture (Jackson, Bittencourt, et al. 2024)) and lightning (e.g., electrical resistivity, thermal conductivity (Gora et al. 2017)), and connecting forest dynamics to storm activity and underlying climate.

New technology provides excellent opportunities to connect storm activity to patterns of tree mortality. Recently launched geostationary satellites (GOES, MTG1) are providing the continuous data necessary to quantify temporal trends in storm activity. Advances in remote sensing methodology could also allow for the quantification of storm‐caused mortality in the historic satellite record, potentially adapting methods already deployed to detect selective logging (e.g., (Welsink et al. 2023)). We also need stronger mechanistic connections between storm characteristics and patterns of tree mortality. Unlike physiological mechanisms of mortality (VPD, temperature, etc.) that are challenging to confirm in the field even when their mortality events are observed, it is comparatively simple to identify wind, lightning, and landslide‐caused mortality in the field, at least if they are observed soon enough after the event (Fontes et al. 2018; Yanoviak et al. 2017). Consequently, we can quantify the connections between satellite‐detected storm characteristics and storm‐caused biomass mortality in the field if we survey sufficient area with adequate frequency. Given how little effort has been invested in studying the effects of storms, there is potential for rapid advances in this field of study.

It is important that future research into the effects of storms captures pantropical variation in both forest taxa and their environment. Like most aspects of tropical forests, the vast majority of data related to storm‐caused disturbance comes from a few places (e.g., Panama, Brazil, and Borneo) (Gora and Yanoviak 2020; Jackson et al. 2021; Silvério et al. 2019; Yanoviak et al. 2017) that are then extrapolated with high uncertainty. However, climate and floristics vary widely among tropical forests (Hagen et al. 2021) and forest responses to climate change appear to differ among regions too (Bennett et al. 2021, 2023; Hubau et al. 2020). Storm activity is also highly variable over space, and temporal trends in storm activity could vary among regions (Gora et al. 2020; Harel and Price 2020; Lavigne et al. 2019). Additionally, interspecific variation in tolerance to wind and lightning suggests that forests could differ in their tolerances to storm‐caused disturbance (Jackson, Bittencourt, et al. 2024; Richards et al. 2022), meaning that differences in floristics could cause patterns of storm‐caused mortality to diverge from spatiotemporal patterns of storm activity. To understand how storms are reshaping tropical forests, we need research investment across broad variation in floristics, climate, and soils.

A complete understanding of storm‐caused mortality requires information about interactions with other agents of tree death. Storms exhibit strong interactions with other agents of mortality, including fire damage increasing wind‐caused mortality risk (Silvério et al. 2019) and lianas increasing tree mortality rates within lightning‐caused disturbances (Gora et al. 2023). Storm‐caused disturbance could also increase tree susceptibility to other factors; for example, lightning facilitates beetle colonisation with uncertain contributions to patterns of tree death (Lawhorn et al. 2025; Parlato et al. 2020). It is likely that many interactions shape patterns of storm‐associated mortality, and therefore understanding the effects of storms requires a deeper understanding of these comorbidities and their spatiotemporal variation. Accordingly, rather than focusing on a single driver of tree death, we need a holistic approach to investigating the patterns and mechanisms underlying tropical forest change.

The consequences of failing to identify the contributions of storms could be substantial. We need to understand the mechanisms by which climate is altering tropical forests, and include those mechanisms in demographic vegetation models, to accurately forecast these forests and future climate (Friend et al. 2014; Koch et al. 2021; Pugh et al. 2019). Moreover, if we miss or misidentify the drivers of forest change, then this could mislead reforestation and forest management efforts that must prioritise taxa that can thrive under future climate. Because the effects of storms on mortality are greatest among canopy trees and mature forests, we may not realise the consequences of misguided reforestation efforts until forests reach maturity decades after intervention. However, if we can identify and quantify the primary climate drivers of forest change, and link them to climate projections, then we can guide forest management practices for long‐term sustainability. Overall, in addition to important efforts to understand factors such as temperature, drought stress, and CO_2_ fertilisation, we need substantial research investment into the contributions of storms to shifting forest dynamics and the weakening of the tropical forest carbon sink.

## 5 Methods

### 5.1 Reanalysis of Plot‐Based Study of Pantropical Biomass Carbon Stocks and Fluxes

The re‐analysis of Sullivan et al. (2020) used the response data and covariates from the published analyses, but added lightning frequency as a proxy for convective storm activity. We used the latitude and longitude of the analysed forest plots to extract lightning frequency values (lightning strikes to the ground per km^2^ per year^−1^) from the data used by Gora et al. (Gora et al. 2020). For Sullivan et al., forest carbon stocks and fluxes were calculated from the ForestPlots.net pantropical network of recensused forest plots, soils data were extracted from the SoilsGrids database, and climate data were extracted from the WorldClim2 database (Sullivan et al. 2020). The lightning frequency data were produced by Earth Networks Total Lightning Network, which is a network of sensors distributed across the Earth's surface that record lightning activity continuously and are specifically designed to detect lightning strikes that contact the Earth's surface (Gora et al. 2020). We used lightning frequency as a proxy for storm activity because it is an excellent indicator of convective storm activity—that is, nearly all lightning occurs in a convective storm—and it is associated with strong convective winds (Williams et al. 1999; Williams 2005).

### 5.2 Estimated Contributions of Storms to Increasing Tree Mortality

We estimated the potential contributions of convective storms to increased biomass mortality using values from the literature. Specifically, we multiplied the range of potential contributions of storms to historic tree biomass mortality, which was based on various literature sources (Esquivel‐Muelbert et al. 2020; Gora et al. 2020, 2021; Gora and Esquivel‐Muelbert 2021; Negrón‐Juárez et al. 2018; Rifai et al. 2016; Yanoviak et al. 2020), by the published range of rates of increasing storm activity over recent decades (Harel and Price 2020; Lavigne et al. 2019) to estimate the percent increase in tree biomass mortality that could result from increases in storm activity. We then compared this trend to the observed increase in tree biomass mortality across Amazonian forest plots from 1990 to 2011 (Brienen et al. 2015).

### 5.3 Comparisons of Storm Activity and Drought Stress

We also evaluated whether spatial variation in storm activity was associated with spatial variation in drought stress. Specifically, we extracted hourly convective available potential energy (CAPE), air pressure at the Earth's surface, and rainfall, air and dewpoint temperature at 2 m above the Earth's surface at 0.25° spatial grain from reanalysis products for the Amazon region, including both Andean and Guiana Shield forests, from 1990 to 2019 (European Centre For Medium‐Range Weather Forecasts 2019). To calculate MCWD, we also extracted monthly precipitation and potential evapotranspiration from the CHELSA database for the same spatial domain and timeframe (Karger et al. 2017, 2018). We focused on this spatial domain because it is the largest and best‐studied region of tropical forests and it is, accordingly, the source of the strongest evidence for tropical forest change. We confirmed that the patterns were unchanged when excluding non‐forest area across this region.

We estimated the average annual convective activity of a given grid cell as the amount of time that grid cell experienced levels of CAPE above a general threshold for convection, averaged over years. We used the 75th quantile of CAPE or 1023 J kg^−1^ for the main text, and two alternative higher thresholds for convection for the Supplemental Figure to confirm that the spatial patterns are consistent regardless of the threshold used. We used CAPE > 1023 J kg^−1^ because this is approximately the threshold above which CAPE is sufficient to produce strong convection, and average afternoon CAPE above 1023 K kg^−1^ is predictive of convective storm activity and spatial patterns of large‐scale windthrow across this spatial domain (Feng et al. 2023).

We estimated maximum annual aridity as mean annual vapour pressure deficit in the driest quarter for each grid cell. After obtaining the hourly reanalysis data, we calculated hourly VPD using Equations 1–5 in Fang et al. (Fang et al. 2022) and then calculated mean VPD for each month. We estimated the peak annual atmospheric drought stress for each grid cell as VPD in the driest quarter of the year (VPD averaged across the three consecutive months in each year with the highest VPD values), which was also averaged over years. We used mean VPD in the driest quarter because it represents atmospheric drought stress and is associated with long‐term trends of increased mortality in Australia (Bauman et al. 2022).

We estimated maximum deficit in ground water supply as the mean annual value of maximum climatological water deficit (Aragão et al. 2007). We calculated monthly water deficit for the entire study area and time period as precipitation minus potential evapotranspiration. Climatological water deficits accumulated across each hydrological year, which was defined as beginning after the month that received the most average rainfall across the 1990–2019 study period (i.e., the water deficit reset each year after the month during which it typically experienced the most rainfall). Maximum climatological water deficit was defined as the largest value of the cumulative water deficit within each calendar year, which was then averaged across years for each grid cell to produce mean annual MCWD. We used MCWD because it is an excellent proxy for ground water supply (Aragão et al. 2007).

## Author Contributions

E.M.G. conceived the study and led the writing of the manuscript. E.M.G., I.R.M. and M.W.C. conducted analyses. All authors contributed to the conceptual development of the study, co‐authored the manuscript, and approved the final version for submission.

## Conflicts of Interest

The authors declare no conflicts of interest.

## Peer Review

The peer review history for this article is available at https://www.webofscience.com/api/gateway/wos/peer‐review/10.1111/ele.70157.

## Supporting information


Data S1.

## Data Availability

All data and code are available on the Cary Institute Figshare: https://doi.org/10.25390/caryinstitute.28925100 (DOI: 10.25390/caryinstitute.28925100).
