# Supplementary material for: Storms Are an Important Driver of Change in Tropical Forests
Source: Ecol Lett. 2025 Jul 9;28(7):e70157. doi: 10.1111/ele.70157 (PMC12272328; doi:10.1111/ele.70157)
Supplement: Supplementary file 1 — Data S1. [file ELE-28-0-s001.pdf]

Supplementary Materials

Storms are an important driver of change in tropical forests

Authors:

Evan M. Gora[1,2*], Ian R. McGregor[1], Helene C. Muller-Landau[2], Jeffrey C. Burchfield[3], KC Cushman[4], Vanessa E. Rubio[1], Gisele Biem Mori[5,6], Martin J. P. Sullivan[7], Matthew W. Chmielewski[8], Adriane Esquivel-Muelbert[9,10]

1. Cary Institute of Ecosystem Studies, Millbrook, New York, NY, USA.

2. Smithsonian Tropical Research Institute, Balboa, Panama

3. Department of Atmospheric Science, University of Alabama in Huntsville, Huntsville, AL, USA.

4. Jet Propulsion Laboratory, California Institute of Technology, Pasadena, CA, USA.

5. National Institute for Amazon Research (INPA), Manaus, Brazil

6. Programa de Pós-graduação em Ecologia e Conservação, Universidade do Estado de Mato Grosso, Nova Xavantina, Brazil

7. Department of Natural Sciences, Manchester Metropolitan University, Manchester, UK

8. Department of Biology, University of Louisville, Louisville, KY, USA.

9. School of Geography, Earth and Environmental Sciences, University of Birmingham, Birmingham, United Kingdom

10. Birmingham Institute of Forest Research, University of Birmingham, Birmingham, United Kingdom

*Corresponding author: email: gorae@caryinstitute.org

Table S1. Model fit statistics and selected coefficients of alternative models for spatial variation in tropical forest carbon. We compare the models presented in the main text of Sullivan *et al.*(2020) - (highlighted in italics) with alternatives that include lightning strike frequency (strikes km^{-2} yr^{-1}) as an additional explanatory variable, include maximum climatological water deficit (MCWD) instead of precipitation in the driest quarter (PrecipDQ) to represent water availability, and/or include additional interaction terms of water availability with maximum temperature (WxT) and lightning frequency with maximum temperature (LxT). The best model for each response variable is highlighted in bold. We used the same methods as Sullivan et al. (2020) to evaluate model fit, model coefficients, and interaction effects. Standardized coefficients were calculated using multi-model inference, including models within 4 AICc of the best model. Model fit is represented by the range of AICc values for the component models used in the multi-model inference analysis and R^2 calculated using the model-averaged coefficients. We present results for models with interaction terms only when they improved model fit (i.e., decreased AICc of the full model). In addition to the variables included here, each model included the 14 other variables used by Sullivan *et al.*(2020): minimum temperature, cloud cover, wind speed, soil texture, soil fertility, continent, and eight spatial eigenvectors. Lightning frequency did not influence carbon gains, so those models are not reported here.

Response variable	Variables			Model fit		Standardized coefficients from multi-model inference (SE)				
	Water Availab.	Lightning frequency	Interactions	AICc range	R^2 (%)	Maximum temperature	Water availability	Lightning frequency	Water x MaxTemp	Lightning x MaxTemp
Carbon stocks	*PrecipDQ*	*Omitted*	*None*	*81.3-84.9*	*43.5*	*-0.089 (0.022)*	*0.045 (0.018)*	-	-	-
		Omitted	*WxT*	*68.5-72.4*	*44.9*	*-0.073 (0.020)*	*0.059 (0.018)*	-	*0.046 (0.011)*	-
		Included	None	64.1-68.0	44.9	-0.074 (0.021)	0.064 (0.018)	-0.049 (0.011)	-	-
		Included	WxT; LxT	50.3-54.3	46.8	-0.053 (0.022)	0.078 (0.019)	-0.049 (0.012)	0.050 (0.012)	-0.029 (0.014)
	**MCWD**	Omitted	None	71.4-75.3	44.4	-0.078 (0.020)	0.065 (0.015)	-	-	-
		Included	None	46.7-50.6	46.5	-0.060 (0.020)	0.088 (0.015)	-0.058 (0.012)	-	-
		**Included**	**WxT; LxT**	**32.2-36.0**	**48.6**	**-0.016 (0.021)**	**0.078 (0.016)**	**-0.062 (0.012)**	**0.047 (0.011)**	**-0.031 (0.013)**
Carbon residence time	*PrecipDQ*	*Omitted*	*None*	*299.2-303.1*	*30.2*	*-0.006 (0.029)*	*0.061 (0.023)*	-	-	-
		Omitted	*WxT*	*286.1-290.0*	*32.1*	*-0.001 (0.027)*	*0.063 (0.023)*	-	*0.056 (0.014)*	-
		Included	None	297.6-301.5	30.6	0.002 (0.029)	0.066 (0.023)	-0.029 (0.015)	-	-
		Included	WxT	285.0-289.0	32.4	0.005 (0.027)	0.069 (0.023)	-0.027 (0.015)	0.056 (0.014)	-
	**MCWD**	Omitted	None	275.6-279.5	32.6	0.006 (0.024)	0.104 (0.018)	-	-	-
		**Included**	**None**	**268.5-272.3**	**33.6**	**0.019 (0.024)**	**0.118 (0.018)**	**-0.046 (0.015)**	**-**	**-**

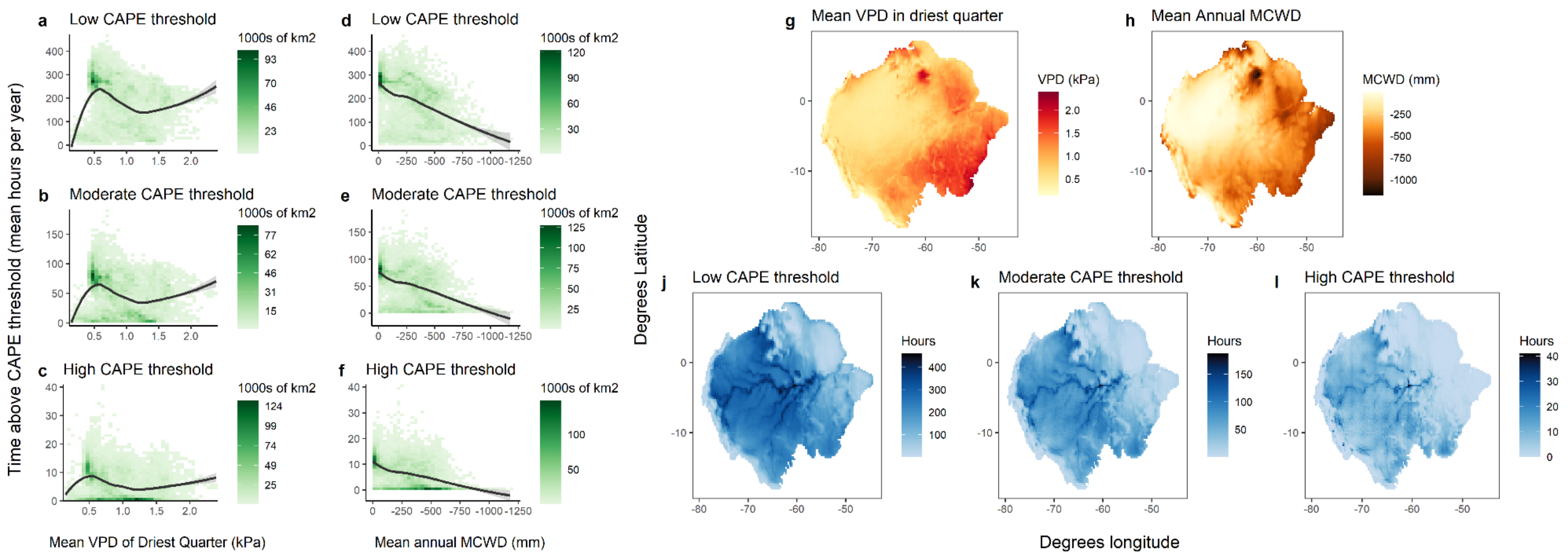

Fig. S1. Storm activity and aridity are generally unrelated across the Amazon. On the left, the amount of time that forests experienced weak (panel a and d; 75th quantile of CAPE or 1023 J kg^{-1}), moderate (panel b and e; 92nd quantile or 1900 J kg^{-1}), or strong (panel c and f; 99.2 quantile or 3000 J kg^{-1}) CAPE exhibited little variation across the range of VPD in the driest quarter of the year (VPD averaged across the three months of highest VPD values) and showed a negative correlation with mean annual MCWD (mm). These values were averaged over the years 1990-2019. On the right, these same trends are shown as maps of the Amazon, including Andean and Guiana Shield forests, with VPD in the driest quarter (panel g), mean annual MCWD (panel h), hours with CAPE above a relatively low threshold (panel j), hours with CAPE above a moderate threshold (panel k), and hours with CAPE above a high threshold (panel l). Shading in panels a-f represent total forest area that experiences those climatic conditions, whereas colors in panels g-l represent variation in each metric over space. We focus this analysis of present-day spatial patterns on the Amazon Basin because this is the region with the strongest evidence of forest change, likely because it is comparatively well-studied. Figure 4 presents panels a, d, g, h, and j.
